# Comparative Analysis of ChatGPT-4o and Gemini Advanced Performance on Diagnostic Radiology In-Training Exams

**DOI:** 10.7759/cureus.80874

**Published:** 2025-03-20

**Authors:** Kian A Huang, Haris K Choudhary, William M Hardin, Neelesh Prakash

**Affiliations:** 1 Radiology, USF Health Morsani College of Medicine, Tampa, USA

**Keywords:** artificial intelligence in radiology, chat gpt, chatgpt-4o, gemini advanced, radiology medical education

## Abstract

Background

The increasing integration of artificial intelligence (AI) in medical education and clinical practice has led to a growing interest in large language models (LLMs) for diagnostic reasoning and training. LLMs have demonstrated potential in interpreting medical text, summarizing findings, and answering radiology-related questions. However, their ability to accurately analyze both written and image-based content in radiology remains uncertain with newer models. This study evaluates the performance of OpenAI's Chat Generative Pre-trained Transformer 4o (ChatGPT-4o) and Google DeepMind's Gemini Advanced on the 2022 ACR (American College of Radiology) Diagnostic Radiology In-Training (DXIT) Exam to assess their capabilities in different radiological subfields.

Methods

ChatGPT-4o and Gemini Advanced were tested on 106 multiple-choice questions from the 2022 DXIT exam, which included both image-based and written-based questions spanning various radiological subspecialties. Their performance was compared using overall accuracy, subfield-specific accuracy, and two-proportion z-tests to determine significant differences.

Results

ChatGPT-4o achieved an overall accuracy of 69.8% (74/106), outperforming Gemini Advanced, which scored 60.4% (64/106), although the difference was not statistically significant (p = 0.151). In image-based questions (n = 64), ChatGPT-4o performed better (57.8%, 37/64) than Gemini Advanced (43.8%, 28/64). For written-based questions (n = 42), ChatGPT-4o and Gemini Advanced demonstrated similar accuracy (88.1% vs. 85.7%). ChatGPT-4o exhibited stronger performance in specific subfields, such as cardiac and nuclear radiology, but neither model showed consistent superiority across all radiology domains.

Conclusion

LLMs show promise in radiology education and diagnostic reasoning, particularly for text-based assessments. However, limitations such as inconsistent responses and lower accuracy in image interpretation highlight the need for further refinement. Future research should focus on improving AI models' reliability, multimodal capabilities, and integration into radiology training programs.

## Introduction

The rapid advancement of artificial intelligence (AI) has led to the development of large language models (LLMs) that are capable of answering complex questions, especially in the medical field. Such LLM models include OpenAI's Chat Generative Pre-trained Transformer 4 (ChatGPT-4), Chat Generative Pre-trained Transformer 4o (ChatGPT-4o), and Google DeepMind's Gemini Advanced, all of which have demonstrated proficiency on standardized exams, including the United States Medical Licensing Examination (USMLE) and specialty board exams. Although most LLMs are proficient in medical examinations, emerging research suggests that these models can often differ in their accuracy, reasoning strategies, and ability to handle medical image interpretation, which makes direct comparisons between these models essential. Several recent studies have highlighted key differences in performance among LLMs. For example, when ChatGPT-4o was compared to Gemini 1.5 Pro on ophthalmology-related board questions, it was found that both models performed well. However, their explanation styles differed, with Gemini 1.5 Pro providing longer justifications for incorrect answers, while ChatGPT-4o gave a more concise response [1]. This means that if users prefer long and detailed explanations, then ​Gemini Advanced should be chosen, but if short explanations are desired, then ChatGPT-4o should be utilized. Between ChatGPT-4 and ChatGPT-4o, ChatGPT-4o is superior in its maximum precision and deep contextual understanding. GPT-4o is better able to strike a balance between its performance and computational efficiency, which makes it better suited for real-time applications in high-demand environments [2]. In an environment such as healthcare, where response time and accessibility are critical, GPT-4o's reduced computational cost and quicker speeds would allow for a much easier and more practical integration for workflows.

A crucial difference between these models is their performance on text-based versus image-based medical questions. When ChatGPT-4o, ChatGPT-4, Gemini 1.5 Pro, and Claude 3 Opus were tested on 790 questions from the Japanese National Medical Examination, it was found that ChatGPT-4o had the highest accuracy rate of 89.2% (n = 705) and had outperformed the other LLMs in both overall performance and each specific category. In imaging-based questions (n = 199), it was found that GPT-4o had an accuracy rate of 80.4% (160), which was superior to Gemini 1.5 Pro's and Claude 3 Opus's accuracy rate of 74.60% (148) and GPT-4's accuracy of 67.30% (n = 134) [3]. Similarly, Silbergleit et al. (2024) assessed ChatGPT-4o and Gemini Advanced on radiology reports and found that ChatGPT-4o had higher consistency in structured interpretation tasks, while Gemini showed more variability [4]. Based on these findings, it appears that ChatGPT-4o is superior to other LLMs in both text-based and image-based questions.

Within the field of radiology, ChatGPT-4 has been tested extensively on many different board-style exams, but its limitations remain a concern. When ChatGPT-4 was tested on the 2022 American College of Radiology (ACR) Diagnostic Radiology In-Training Examination (DXIT) (n = 106), it was found that ChatGPT-4 achieved an overall accuracy of 58.5% (n = 62), which was comparable to a second-year radiology resident but inferior to a third-year resident. The study showed that ChatGPT-4 especially struggled with image-based questions (n = 64), where it was only able to score 45.4% (n = 29) compared to 80.0% (n = 34) on text-based questions (n = 42), suggesting weaknesses in GPT-4's radiologic image interpretation [5].

Despite these findings, ChatGPT-4o and Gemini Advanced have not been evaluated on the ACR DXIT exam, leaving a critical gap in understanding how newer LLMs compare in a structured radiology assessment. This study aims to systematically compare ChatGPT-4o and Gemini Advanced. By evaluating multiple LLMs across both text-based and image-based diagnostic reasoning, this study will provide critical insights into the strengths and limitations of general-purpose AI models in clinical radiology. These findings will inform the potential role of LLMs in radiology training, clinical decision support, and future AI-assisted diagnostic workflows.

## Materials and methods

The 2022 July ACR DXIT exam (total questions, n = 106) containing both image-based (n = 64) and written-based questions (n = 42) was administered to each LLM. Permission was obtained from the ACR to utilize DXIT exam materials for testing purposes. Using a similar methodology from Payne et al. (2024) [5], each LLM was administered a brief standardized prompt prior to the exam: "Imagine you are a radiology resident taking the Diagnostic Radiology In-Training exam. Answer these questions to the best of your ability. Do you understand?" Questions and multiple-choice answers were inputted simultaneously, along with any associated images that accompanied question stems. All questions were asked in the order presented on the exam, and each LLM completed the entire set in a single session. Response length was not explicitly capped to allow for natural variation in answer explanations. Additionally, images were kept in their native resolution and copied directly from the exam to preserve diagnostic quality.

Performance metrics include overall question accuracy, image-based question accuracy, written-based question accuracy, and subtopic question accuracy (neuroradiology, pediatric radiology, musculoskeletal, etc.), which were collected for each LLM. To assess differences in accuracy, a two-proportion z-test was used for each performance category. This statistical test compares the proportions of correct answers between two independent groups (ChatGPT-4o and Gemini Advanced) to determine if the observed differences are statistically significant. All statistical analyses were carried out using Python 3.11 (Python Software Foundation, Fredericksburg, VA), and a p-value < 0.05 was considered significant.

## Results

**Figure 1:**
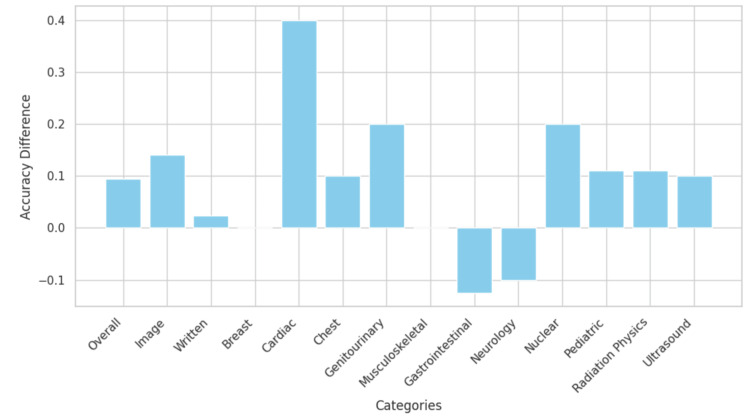
Accuracy differences between ChatGPT-4o and Gemini Advanced Data have been represented as differences in accuracy between ChatGPT-4o and Gemini Advanced (ChatGPT-4o Accuracy - Gemini Advanced Accuracy) among various radiology subfields in the DXIT exam, with positive values favoring ChatGPT-4o and negative values favoring Gemini Advanced. Non-existent bars demonstrate an accuracy difference of 0 between each LLM.

The performance of ChatGPT-4o and Gemini Advanced on the 2022 ACR DXIT exam was evaluated across various metrics, including overall accuracy, image-based question accuracy, written-based question accuracy, and subtopic-specific accuracy. As shown in Table 1, ChatGPT-4o achieved an overall accuracy of 69.8% (n = 74), outperforming Gemini Advanced, which scored 60.4% (n = 64). However, using a two-proportion z-test, this difference was not statistically significant (p = 0.151). Regarding image-based (n = 64) and written-based questions (n = 42), ChatGPT-4o scored 57.8% (n = 37) and 88.1% (n = 37), respectively, while Gemini Advanced scored 43.8% (n = 28) and 85.7% (n = 36), respectively (Table 1). The z-test did not indicate statistical significance for either of these comparisons (p = 0.113, p = 0.744, respectively). ChatGPT-4o outperformed Gemini in most radiological subtopics, with a few topics showing mixed or comparable performance levels. The full results are presented in Table 1. Figure 1 presents the accuracy differences for each performance category, with positive values indicating better performance favoring ChatGPT-4o and negative values favoring Gemini Advanced. Statistical testing across categories showed that differences in accuracy between the two models did not reach statistical significance (p > 0.05 for all comparisons).

**Table 1:** Two-proportion Z-tests Two-proportion z-tests were calculated for each performance category between ChatGPT-4o and Gemini Advanced. Data have been represented as the accuracy of correct answers (%), question count (n), two-proportion z-statistic, and its associated p-value. A p-value < 0.05 was considered statistically significant.

Category	ChatGPT-4o Accuracy (%)	Gemini Accuracy (%)	Questions (n)	Z-statistic	p-value
Overall	69.8	60.4	106	1.436	0.151
Image	57.8	43.8	64	1.584	0.113
Written	88.1	85.7	42	0.326	0.744
Breast	60	60	10	0.000	1.000
Cardiac	70	30	10	1.789	0.074
Chest	90	80	10	0.626	0.531
Genitourinary	60	40	10	0.894	0.371
Musculoskeletal	50	50	10	0.000	1.000
Gastrointestinal	50	62.5	8	-0.504	0.614
Neurology	80	90	10	-0.626	0.531
Nuclear	80	60	10	0.976	0.329
Pediatric	44.4	33.3	9	0.483	0.629
Radiation Physics	88.9	77.8	9	0.632	0.527
Ultrasound	90	80	10	0.626	0.531

## Discussion

The results of this study demonstrate that ChatGPT-4o outperforms Gemini Advanced in overall accuracy (n = 106), 69.8% (n = 74) vs. 60.4% (n = 64). There was a notable advantage in image-based questions (n = 64), 57.8% (n = 37) vs. 43.8% (n = 28), respectively, and several radiology subcategories, including cardiac and nuclear radiology. However, both models performed similarly in written-based questions (n = 42), where ChatGPT-4o achieved an accuracy of 88.1% (n = 37) compared to 85.7% (n = 36) for Gemini Advanced. Despite these observed differences, statistical analyses did not reveal significant differences (p > 0.05), suggesting that the variations could be due to chance rather than a fundamental difference in model capability. Additionally, these findings highlight the challenge of model drift, where updates or retraining may alter performance unpredictably over time, impacting consistency in medical applications. These findings align with previous research, such as Bera et al.'s study on GPT-4's performance in radiology board-style exams, where the model demonstrated strengths in cardiovascular imaging but struggled with thoracic imaging and overall reproducibility [6]. Using the same DXIT exam set, previous research by Payne et al. tested ChatGPT-4 (the preceding version of 4o) and found a 58.5% (n = 62) overall accuracy, which fell between first-year radiology resident (52.8%) and second-year radiology resident performance (61.9%) [5]. Comparatively, our study found that ChatGPT-4o surpassed second-year radiology resident performance with an overall accuracy of 69.8%, whereas Gemini Advanced had a more similar accuracy to ChatGPT-4 (60.4%). However, given the evolving nature of LLMs, these accuracy levels may shift with future updates, affecting their generalizability across different time points and datasets.

Our study also found that ChatGPT-4o changed 24.5% (n = 26) of its previous answers at a six-month follow-up of repeated exam administration. This is similar to the study by Payne et al., which found that ChatGPT-4 had a 25.5% (n = 27) answer variance at follow-up, potentially indicating an intentional mechanism within the model to naturally vary or fluctuate answers or reasoning to achieve such answers [5]. Such variability over time raises concerns about long-term reliability and the potential need for periodic recalibration when deploying LLMs in medical education and diagnostics. A six-month follow-up using Gemini Advanced failed due to the model not accepting and analyzing most of the DXIT medical imaging questions for reasons that were not disclosed.

These findings have important implications for the role of evolving LLMs in radiology and AI-assisted diagnostics. The performance of both models suggests that LLMs have the potential to serve as supplementary tools in radiology education and diagnostic reasoning. Their high accuracy in written-based questions indicates a strong comprehension of medical text, which could be useful for interpreting radiological reports and aiding trainees. However, the lower accuracy in image-based questions highlights a major limitation in current AI models, as radiology relies heavily on image interpretation. Prior studies have also noted that GPT-4 struggles with visual data interpretation, suggesting that further advancements in multimodal AI models are necessary before LLMs can reliably assist with complex radiological diagnostics [7,8]. Moreover, the observed inconsistencies in accuracy across different radiology subfields suggest limitations in generalizability, as model performance may depend on the specific nature of the dataset on which it was trained.

Despite their potential, LLMs also present challenges and limitations. A key concern is the inconsistency of responses, as previous research has shown that GPT-4 can produce different answers to the same question in separate test runs, raising concerns about its reliability [5,6]. Furthermore, while ChatGPT-4o generally performed better than Gemini Advanced, performance varied across radiology subtopics, with no model consistently excelling across all domains. This variability underscores the importance of external validation and careful implementation when integrating LLMs into clinical workflows. This aligns with previous findings that demonstrated variability in AI accuracy depending on the specific type of radiological case and the source of information used for training.

Comparing the results of this study with prior research, the performance of GPT-4o appears to be in line with previous assessments of AI in radiology. Studies evaluating ChatGPT-4 and its predecessors have found that AI models perform comparably to human trainees in some areas but struggle in highly specialized diagnostic tasks [5,9,10]. For example, in musculoskeletal and neuroradiology, previous studies have shown that GPT-4's diagnostic accuracy is lower than that of board-certified radiologists, although it performs similarly to radiology residents [9,10]. These findings suggest that while LLMs have the potential to support radiologists, they are not yet at a level where they can replace expert human judgment. Future research should focus on optimizing multimodal AI models custom-trained to radiology datasets to improve image interpretation capabilities, as this remains a key limitation of current LLMs. Additionally, strategies to mitigate model drift, such as fine-tuning models with domain-specific datasets and implementing consistency checks, will be critical to ensuring sustained accuracy in radiology applications. Another important area for future development is the integration of AI into radiology education. As LLMs improve in performance (such as from ChatGPT-4 to ChatGPT-4o), they could potentially serve as valuable tools for training radiology residents by simulating board-style exams or providing real-time feedback on diagnostic cases.

## Conclusions

This study provides insights into the diagnostic capabilities of ChatGPT-4o and Gemini Advanced in radiology-based assessments. While ChatGPT-4o demonstrated higher accuracy than Gemini Advanced, the lack of statistically significant differences suggests that both models remain limited in their application to radiological diagnostics. Their strengths in written-based questions indicate potential utility in educational settings, but their performance in image-based tasks remains suboptimal. The findings emphasize the need for continued AI development, particularly in multimodal learning, to enhance radiology-specific applications. Future studies should explore tailored multimodal AI training using radiology datasets and investigate their role in assisting early radiology residents with exam questions and potentially educational case studies.

## Appendices

**Table 2:** Exam answer key and large language model responses Data are presented as multiple-choice responses from each large language model in the Diagnostic Radiology In-Training exam. Questions are categorized by topic and associated with a correct answer sourced from the exam answer key.

Question Number	ChatGPT-4o Answer	Gemini Advanced Answer	Question Type	Correct Answers
1	D	D	Breast	D
2	C	C	Breast	A
3	B	A	Breast	B
4	A	A	Breast	B
5	B	B	Breast	B
6	C	C	Breast	C
7	B	D	Breast	D
8	D	A	Breast	C
9	B	B	Breast	B
10	D	D	Breast	D
11	C	B	Cardiac	C
12	D	A	Cardiac	D
13	A	B	Cardiac	C
14	D	D	Cardiac	D
15	A	B	Cardiac	A
16	C	A	Cardiac	B
17	D	D	Cardiac	D
18	A	C	Cardiac	D
19	A	A	Cardiac	A
20	D	B	Cardiac	D
21	B	B	Chest	A
22	B	B	Chest	B
23	B	B	Chest	B
24	D	D	Chest	D
25	C	C	Chest	C
26	A	A	Chest	A
27	D	C	Chest	D
28	B	B	Chest	B
29	C	C	Chest	C
30	A	A	Chest	A
31	C	C	Genitourinary	D
32	C	C	Genitourinary	A
33	D	C	Genitourinary	D
34	D	D	Genitourinary	D
35	C	C	Genitourinary	C
36	A	A	Genitourinary	B
37	A	A	Genitourinary	A
38	A	B	Genitourinary	A
39	C	C	Genitourinary	C
40	B	B	Genitourinary	A
41	D	D	Musculoskeletal	D
42	C	C	Musculoskeletal	C
43	C	D	Musculoskeletal	C
44	D	A	Musculoskeletal	C
45	D	D	Musculoskeletal	C
46	A	A	Musculoskeletal	A
47	A	B	Musculoskeletal	A
48	B	B	Musculoskeletal	C
49	B	D	Musculoskeletal	D
50	B	D	Musculoskeletal	D
51	B	B	Gastrointestinal	B
52	A	A	Gastrointestinal	A
53	D	D	Gastrointestinal	B
54	A	C	Gastrointestinal	B
55	A	A	Gastrointestinal	A
56	D	A	Gastrointestinal	A
57	A	B	Gastrointestinal	B
58	D	B	Gastrointestinal	D
59	C	C	Neurology	C
60	B	B	Neurology	B
61	B	B	Neurology	B
62	A	A	Neurology	A
63	D	A	Neurology	D
64	D	C	Neurology	C
65	C	C	Neurology	C
66	A	B	Neurology	B
67	A	A	Neurology	A
68	D	D	Neurology	D
69	B	A	Nuclear	B
70	C	C	Nuclear	C
71	C	C	Nuclear	C
72	B	A	Nuclear	B
73	A	A	Nuclear	A
74	D	D	Nuclear	D
75	C	B	Nuclear	A
76	A	A	Nuclear	A
77	C	C	Nuclear	C
78	A	C	Nuclear	B
79	A	A	Pediatric	B
80	B	A	Pediatric	C
81	A	A	Pediatric	A
82	D	D	Pediatric	B
83	C	D	Pediatric	B
84	A	A	Pediatric	A
85	C	A	Pediatric	C
86	A	B	Pediatric	C
87	B	B	Pediatric	B
88	D	D	Physics	D
89	C	C	Physics	C
90	A	A	Physics	A
91	D	D	Physics	D
92	B	A	Physics	B
93	A	A	Physics	A
94	C	B	Physics	A
95	B	B	Physics	B
96	A	A	Physics	A
97	B	A	Ultrasound	D
98	C	C	Ultrasound	C
99	A	A	Ultrasound	A
100	A	D	Ultrasound	A
101	C	C	Ultrasound	C
102	C	C	Ultrasound	C
103	D	D	Ultrasound	D
104	B	B	Ultrasound	B
105	C	C	Ultrasound	C
106	C	C	Ultrasound	C
